# Radiosensitivity of human tumour cells is correlated with the induction but not with the repair of DNA double-strand breaks

**DOI:** 10.1038/sj.bjc.6601133

**Published:** 2003-07-29

**Authors:** R A El-Awady, E Dikomey, J Dahm-Daphi

**Affiliations:** 1 Department of Radiotherapy and Radiation Oncology, University Hospital of Hamburg-Eppendorf, Martinistr. 52, 20246 Hamburg, Germany; 2 Institute of Biophysics and Radiobiology, University Hospital of Hamburg-Eppendorf, Martinistr. 52, 20246 Hamburg, Germany

**Keywords:** tumour, MCF-7, RT112, Du145, LNCaP, T47D, HeLa, DSB, graded-field gel electrophoresis

## Abstract

Nine human tumour cell lines (four mammary, one bladder, two prostate, one cervical, and one squamous cell carcinoma) were studied as to whether cellular radiosensitivity is related to the number of initial or residual double-strand breaks (dsb). Cellular sensitivity was measured by colony assay and dsb by means of constant- and graded-field gel electrophoresis (CFGE and GFGE, respectively). The nine tumour cell lines showed a broad variation in cellular sensitivity (SF2 0.17–0.63). The number of initial dsb as measured by GFGE ranged between 14 and 27 dsb/Gy/diploid DNA content. In contrast, normal fibroblasts raised from skin biopsies of seven individuals showed only a marginal variation with 18–20 dsb/Gy/diploid DNA content. For eight of the nine tumour cell lines, there was a significant correlation between the number of initial dsb and the cellular radiosensitivity. The tumour cells showed a broad variation in the amount of dsb measured 24 h after irradiation by CFGE, which, however, was not correlated with the cellular sensitivity. This residual damage was found to be influenced not only by the actual number of residual dsb, but also by apoptosis and cell cycle progression which had impact on CFGE measurements. Some cell line strains were able to proliferate even after exposure to 150 Gy while others were found to degrade their DNA. Our results suggest that for tumour cells, in contrast to normal cells, the variation in sensitivity is mainly determined by differences in the initial number of dsb induced.

Cancer cells exhibit characteristics that distinguish them from their normal counterparts. Three cellular functions tend to be inappropriately regulated in a neoplasm. First, the normal constraints on cellular proliferation are relaxed. Second, differentiation can be distorted. Third, chromosomal and genetic organisation may be destabilised such that variant cells arise with high frequency. All those factors do not only determine the growth and malignant characteristics of the tumour but also their responsiveness to radiation. This ‘intrinsic’ radiosensitivity differs largely between tumour types and is at least partly due to the different sensitivity of the respective tumour cells Deacon *et al*, 1984; Fertil and Malaise, 1985). It was further shown that within one type of tumours, the outcome of the individual patients after radiotherapy was reflected by the *in vitro* radiosensitivity (SF2) of their tumour cells (West *et al*, 1993, 1995; Stausbol-Gron and Overgaard, 1999; Björk-Eriksson *et al*, 2000).

It is generally accepted that among the DNA damage induced mainly double-strand breaks (dsb) (Bryant, 1984; Ward, 1988; Wurm *et al*, 1994; Dikomey *et al*, 1998; Pfeiffer *et al*, 2000), and particularly residual dsb (Dikomey *et al*, 1998, 2000) are responsible for cell killing by ionising radiation. The number of residual dsb depends on both, the number of dsb induced and on the respective repair capacity. In normal cells, no variation was found for the number of induced dsb (Dikomey *et al*, 1998, 2000), illustrating that differences are only due to variations in the repair capacity.

This picture appears to be much more complex and even contradictory for tumour cells. 11 out of 13 studies reported on a variation in the number of induced dsb (Kelland *et al*, 1988; Schwartz *et al*, 1988, 1990, 1991; McMillan *et al*, 1990; Giaccia *et al*, 1992; Olive *et al*, 1994; Ruiz de Almodovar *et al*, 1994; Zaffaroni *et al*, 1994; McKay and Kefford, 1995; Whitaker *et al*, 1995; Woudstra *et al*, 1998; Eastham *et al*, 2001), but only three found a correlation with cell killing (McMillan *et al*, 1990; Ruiz de Almodovar *et al*, 1994; Whitaker *et al*, 1995). In repair studies, a correlation between cell killing and the number of dsb was only found after short repair intervals (up to 2 h) (Schwartz *et al*, 1988, 1990; Giaccia *et al*, 1992; Zaffaroni *et al*, 1994), but never for nonreparable dsb.

These heterogeneous results may be partly explained by the techniques used (see also McMillan *et al*, 2001). Several studies raised doubts as to whether neutral filter elution exclusively detects dsb (Hutchinson, 1989; Okayasu and Iliakis, 1989; Wlodek and Olive, 1990). Pulsed-field gel electrophoresis (PFGE), currently most widely used, has the advantage to resolve large DNA fragments according to the molecular size permitting, in principle, a direct quantification of dsb. However, the accurate analysis of the profile of the continuous fragment distribution is not trivial (Ager *et al*, 1990; Kraxenberger *et al*, 1994; Cedervall *et al*, 1995). In addition, PFGE could be complicated by paradoxical migration patterns (Carle *et al*, 1986; Chu, 1991; Löbrich *et al*, 1993). Therefore, PFGE and constant-field gel electrophoresis (CFGE) are preferably used to quantify only the fraction of DNA fragments released (FDR) from the bulk DNA (Blöcher *et al*, 1989; Stamato and Denko, 1990; Iliakis *et al*, 1991a, 1991b). All three methods are sensitive to replication (Okayasu *et al*, 1988; Stamato and Denko, 1990; Iliakis *et al*, 1991a, 1991b; Dahm-Daphi and Dikomey, 1995), which might be important for continuously proliferating tumour cells.

To this end, we introduced a modification of the standard electrophoresis termed ‘graded-field gel electrophoresis’ (GFGE) (Dahm-Daphi and Dikomey, 1995, 1996; Zhou *et al*, 1997a, 1997b). GFGE operates at stepwise increased electric field strength resulting in distinct bands that contain fragments of different molecular weights. The analysis then permits direct calculation of the number of dsb independent of the cell cycle distribution.

Here we applied the GFGE to determine the number of dsb induced in nine tumour cell lines and for control in seven normal fibroblast lines and compared it with the respective data obtained by CFGE. We further measured the kinetics of dsb repair and the residual damage 24 h after irradiation in order to clarify, whether dsb critically determine tumour cell survival.

## MATERIALS AND METHODS

### Cell lines and culture conditions

The LNCaP and DU145 cell lines were originally isolated from metastatic lesions of patients suffering from prostate cancer (Stone *et al*, 1978; Horoszewicz *et al*, 1983) and purchased from DSMZ (Braunschweig, Germany) as American Type Culture Collection (ATCC) cell lines. HeLa cells were derived from a cervical carcinoma (Scherer *et al*, 1953) obtained from Dr Aubee. The T47D-B8 and MCF-7 cell lines descended from human breast carcinomas (Soule *et al*, 1973; Soto *et al*, 1986; Ruiz de Almodovar *et al*, 1994). The MCF-7 subclones BB and Bus were described to differ from MCF-7 parental cells with respect to dsb induction and repair, p53 status, cell cycle and apoptosis (Nunez *et al*, 1995; Siles *et al*, 1995). These cells as well as the RT112 bladder carcinoma cell line (Masters *et al*, 1986) were gifted by Dr Ruiz de Almodevar. SCC4451 cells were established from a squamous cell carcinoma of the head and neck obtained from Dr Zölzer (Zölzer *et al*, 1995). All cells were either grown in RMPI 1640 or DMEM (Life Science Technology/BRL, Karlsruhe, Germany) supplemented with 10% FCS and penicillin/streptomycin in 5% CO_2_.

Fibroblast cells, HF-1, -2, -7, -8, -46, -60 and HF, were established from punch biopsies as described elsewhere (Borgmann *et al*, 2002). CHO cells were kept in α-MEM medium. The experiments were performed with nearly confluent tumour cells and confluent fibroblasts.

### Clonogenic cell survival

Tumour cell survival was assessed by colony formation assay. Near-confluent cultures were X-irradiated at 220 kVp, at a dose-rate of 2 Gy min^−1^. Irradiated cells were immediately plated and grown for 2–3 weeks with one medium change. Stained colonies of more than 50 cells were counted. Experiments were repeated thrice with three replicates each.

### Constant-field gel electrophoresis

Subconfluent cells were suspended (3 × 10^6^ ml in 0.8% low melting point agarose (Bio-Rad, Munich, Germany) and pipetted into 180 *μ*l plug moulds to solidify (Dahm-Daphi and Dikomey, 1995). Those agarose plugs were irradiated on ice to prevent DNA repair during irradiation and thereafter directly subjected to cell lysis (0.4 M EDTA, 2% *N*-lauryl sarcosine, and 1 mg ml^−1^ proteinase K, all Sigma, Deisenhofen, Germany). Lysis was started on ice for 30 min and continued at 37°C overnight. The plugs were washed thrice with Tris-EDTA buffer and sliced into pieces containing about 10^5^ cells, which were inserted into a 14 × 20 cm 0.8% agarose gel (High-grade ultrapure, Bio-Rad). The gel was then covered with a thin overlayer of 0.8% agarose to avoid light fraction artefacts upon optical imaging. Electrophoresis was performed at 0.6 V cm^−1^ for 30 h in 0.5 × TBE buffer (45 mM Tris base, 45 mM boric acid, 2 mM EDTA) in a conventional apparatus (Subcell, Bio-Rad). The gel was then stained overnight (0.5 *μ*g ml^−1^ ethidium bromide), destained overnight in ddH_2_O and CCD camera (Sony XC-75CE) equipped with an image analysis system (Optimas, Silverspring, MD, USA) was used to quantify the FDR.

### Graded-field gel electrophoresis

Cells were treated as before but electrophoresis was performed differently. Running conditions were now 0.6 V cm^−1^ for 30 h followed by 1.5 V cm^−1^ for 6 h. Compared to the regimen published previously (Dahm-Daphi and Dikomey, 1995) the present protocol was restricted to two different field strengths sufficient for the current purpose. Detailed analysis and mathematics were described elsewhere (Dahm-Daphi and Dikomey, 1995). In principle, analysis was based on an equation given by Blöcher (1990) describing the sigmoid curve of the total FDR obtained by PFGE. Here, the total FDR corresponds to the sum of the FDR of the two bands (GFGE at 0.6 and 1.5 V cm^−1^, Figure 2). The curves of total FDR and FDR at 1.5 V cm^−1^ were both sigmoid (Figure 4) and could, hence, be fitted by the equation described by Blöcher (1990). The third curve (band 1) corresponds to the difference between both fits. The *χ*^2^ fits were calculated simultaneously and gave the number of dsb in unirradiated cells, of dsb induced per DNA-unit, the maximum DNA fragment size in each band and the factor of retention (*f*_ret_).

### Cell cycle analysis

Cell cycle distribution of PI-stained cells was determined by flow cytometry in an FACScan (Becton Dickenson, Heidelberg, Germany).

### Statistics

Each experiment was repeated three times and the data were given as a mean with its standard error (±s.e.m.). Statistical analysis, data fitting and graphics were performed by means of the Prism 3.1 computer program (GraphPad Software, San Diego, USA).

## RESULTS

**Figure 1:**
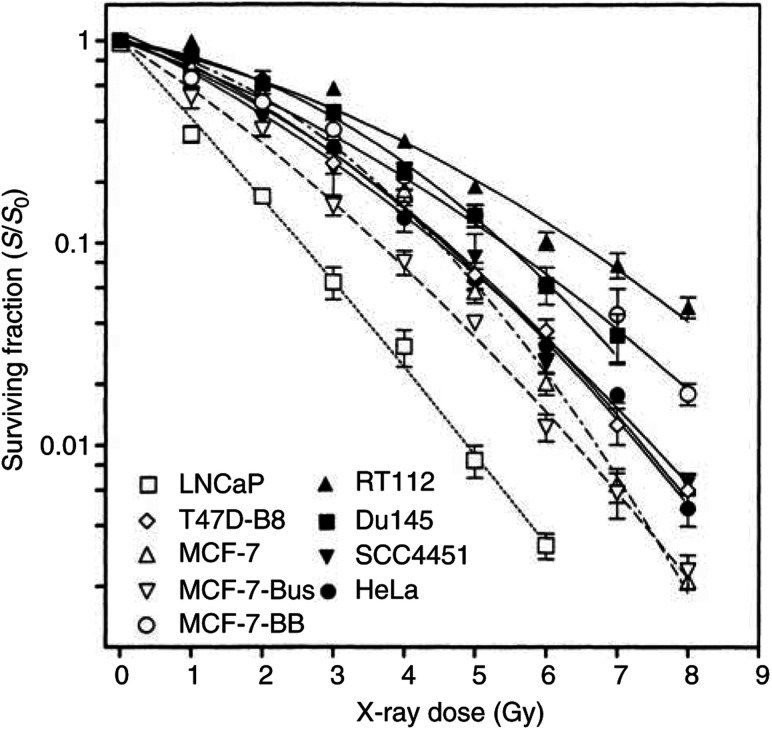
Cellular radiosensitivity of human tumour cell lines. Cell survival was determined by means of colony formation assay. Data were fitted to the linear-quadratic equation −ln(*S*/*S*_0_)=*αD*+*βD*^2^ where *S* is the surviving fraction, *S*_0_ the plating efficiency of unirradiated cells and *D* the X-ray dose.

The nine tumour cell lines showed a wide range of radiosensitivity (Figure 1

). The SF2, the linear-quadratic parameters *α* and *β* and the mean inactivation dose, *D*_bar_ (Fertil *et al*, 1984), were calculated (Table 1

**Table 1:** Parameters of cellular radiosensitivity and cell cycle distribution

					**0 Gy**			**150 Gy**		
**Cell lines**	**SF2^a^**	***α* (Gy^−1^)^b^**	***β* (Gy^−2^)^b^**	***D*_bar_ (Gy)^c^**	**G^d^_1_**	**S**	**G_2_/M**	**G_1_**	**S**	**G_2_/M**
RT112	0.62±0.02	0.18±0.06	0.028±0.007	3.2±0.5	55	33	13	43	54	2
DU145	0.63±0.05	0.12±0.02	0.055±0.005	2.85±0.3	54	36	10	47	44	9
SSC4451	0.41±0.07	0.38±0.15	0.032±0.018	2.04±0.5	80	11	9	83	10	7
HeLa	0.43±0.03	0.33±0.06	0.043±0.007	2.1±0.2	70	18	12	43	40	17
LNCaP	0.17±0.01	0.87±0.06	0.014±0.011	1.11±0.1	78	15	7	68	23	9
T47D-B8	0.46±0.06	0.29±0.4	0.045±0.006	2.21±0.2	65	26	9	53	14	33
MCF-7	0.53±0.06	0.16±0.04	0.078±0.006	2.35±0.3	73	16	11	60	25	15
MCF7-BB	0.51±0.05	0.28±0.03	0.027±0.005	2.55±0.1	47	43	10	70	16	14
MCF7-Bus	0.31±0.03	0.52±0.03	0.03±0.005	1.64±0.1	76	14	10	52	3	13

a Surviving fraction for a X-ray dose of 2 Gy.

b Linear and quadratic term of the dose response.

c D_bar_, mean inactivation dose.

d Percentage of cells in G_1_, S or G_2_M-phase 24 h after 0 or 150 Gy.

). The *D*_bar_ of the most radiosensitive cell line (LNCaP) was 2.9 times lower as compared to the most radioresistant strain (RT112).

### Induction of dsbs

Figure 2

**Figure 2:**
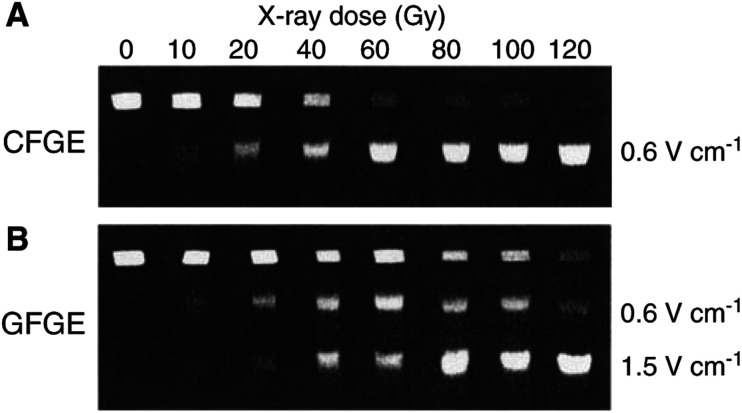
Constant- and graded-field gel electrophoresis, CFGE (upper) and GFGE (lower). CFGE was run at 0.6 V cm^−1^ for 30 h and GFGE at two field strengths of 0.6 V cm^−1^ for 30 h followed by 1.5 V cm^−1^ for 6 h. The fluorescence intensity of each band in each lane was recorded by a CCD video camera.

shows dose-dependent separation of radiation-induced DNA fragments for MCF7-BB cells using CFGE (A) and GFGE (B). CFGE was run at a constant field strength of 0.6 V cm^−1^ collecting all released DNA fragments in a single band. The FDR, as determined by CFGE for all tumour cell lines (Figure 3

**Figure 3:**
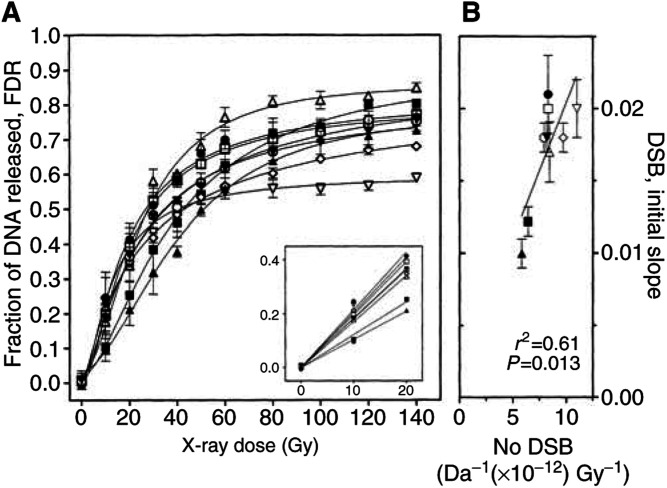
(**A**) Induction of dsb measured by CFGE. Cells were irradiated on ice with doses up to 140 Gy immediately followed by the measurement of dsb by CFGE. The increase of FDR with dose was fitted by nonlinear regression. The initial slope of FDR was determined by linear regression of the data obtained for doses up to 20 Gy (insrt). (**B**) Correlation between the number of dsb induced, as calculated from Figure 4, and the initial slope, as taken from Figure 3A. The symbols correspond to those in Figure 1.

), was found to increase with dose finally reaching a plateau at doses of 100–140 Gy. Clear differences were obvious for both the initial slope (see inset) and the final plateau. The initial slope varied by a factor of 2 (Table 2

**Table 2:** Parameters of initial and residual dsb for tumour and normal cells

**Cell lines**	**Initial slope^a^ (Gy^−1^)**	***k*^b^ 10^−12^ (Gy^−1^ Da^−1^)**	***f*_ret_^c^ (%)**	**N_24 h_(150 Gy)^d^ (Gy-equival.)**
Tumour cell lines				
RT112	0.010±0.001	5.75±0.77	33±3	37.5±4.9
DU145	0.012±0.001	6.39±0.64	30±4	24.1±4.2
SSC4451	0.018±0.001	8.18±0.63	23±4	9.9±2.4
HeLa	0.021±0.003	8.31±0.72	11±3	>150
LNCaP	0.020±0.001	8.31±0.76	12±2	10.2±2.5
T47D-B8	0.018±0.001	9.71±0.70	28±4	>150
MCF-7	0.017±0.002	8.43±0.71	16±3	12.8±1.8
MCF7-BB	0.018±0.001	7.92±0.59	19±3	14.5±2.3
MCF7-Bus	0.020±0.002	11.00±0.65	40±5	>150
*Mean*		*8.22*±*1.6*		
				
Normal human Fibroblasts				
HF-1	Nd^e^	7.76±0.71	3±2	Nd
HF-46	Nd	7.80±0.63	22±3	Nd
HF-2	Nd	8.43±0.69	15±3	Nd
HF-60	Nd	8.05±0.72	17±2	Nd
HF-F	Nd	8.69±0.68	20±5	Nd
HF-8	Nd	7.16±0.75	6±3	Nd
HF-7	Nd	7.54±0.60	6±3	Nd
*Mean*		*7.9*±*0.50*		

a Initial slope calculated by linear regression from data shown in the inset of Figure 3.

b Number of dsb per Gy and per Da calculated from the data plotted in Figure 4.

c Fraction of fragments that did not migrate to band 1 because of being retained in the plug.

d Number of dsb measured 24 h after irradiation with 150 Gy expressed in Gy-equivalents.

e Not determined.

, 1st row) and the final plateau by 1.6.

These data suggested that the nine tumour strains studied differ in the number of dsb induced. However, the absolute number cannot be deduced from these data. Therefore, induction was also measured by GFGE, as previously described (Dahm-Daphi and Dikomey, 1995). Electrophoresis was run at two field strengths, which resulted in two distinct bands containing DNA fragments of different molecular weights (Figure 2B). GFGE hence allows to calculate the absolute number of dsb induced in each cell line. Fractions of DNA released (FDR) to bands 1 and 2 together with the total DNA released were measured for all nine tumour and also for seven human fibroblast lines (Figure 4

**Figure 4:**
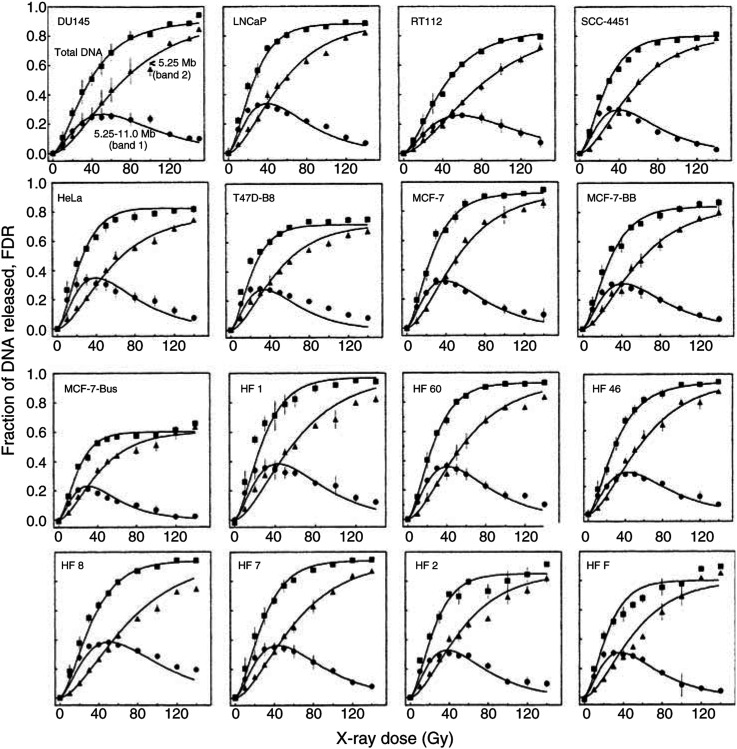
Induction of dsb measured by GFGE. Cells were irradiated on ice with doses up to 140 Gy immediately followed by the measurement of dsb by GFGE. GFGE was run for nine tumour and seven primary fibroblast strains. For each cell line, the FDR of band 1 (circles), band 2 (triangles) and the sum of both (squares) were commonly fitted by nonlinear regression (see text and Dahm-Daphi and Dikomey, 1995).

). In all cases, FDR of band 1 first increased with the dose, reaching a maximum at a certain dose and declined thereafter. In contrast, band 2 increased continuously. The fraction of total DNA released, which is the sum of FDR in bands 1 and 2, gave a similar curve to that of CFGE (Figure 3). Bands 1 and 2 differed considerably between the cell lines, which was most obvious for the dose at which band 1 reached its maximum. For example, RT112 and DU145 cells band 1 reached this maximum at doses of 55–60 Gy, in contrast to a two-fold lower dose of 30 Gy measured for MCF-7-Bus cells.

For each strain, the FDR in bands 1 and 2 were simultaneously fitted as function of dose using a model previously described in detail (Dahm-Daphi and Dikomey, 1995). Those fits gave the number of dsb and a retention factor (*f*_ret_). The comparison of all strains gave by fitting the size of the fragments in each band. According to these fits band 1 contained DNA fragments of 5.25–11.0 Mbp and band 2 of less than 5.25 Mbp. Theses values are slightly higher than previously reported (Dahm-Daphi and Dikomey, 1995). Now, 16 different cell lines have been analysed instead of only two data sets before (Dahm-Daphi and Dikomey, 1995). The number of dsb induced per Gy and Da was found to vary by a factor of 2 between 5.75 for RT112 and 11.5 × 10^−12^ dsb/Gy/Da for MCF7-Bus (Table 2). The respective range for the seven normal fibroblast lines was significantly smaller (7.2–8.6 × 10^−12^ dsb/Gy/Da). These values are equivalent to a variation of 14–27 dsb/Gy/diploid DNA content for tumour cells and 18–20 dsb/Gy/diploid DNA content for normal fibroblasts.

Figure 3B shows the relationship between the initial slope of the FDR curves measured by CFGE (Figure 3A) and the number of induced dsb as obtained from GFGE (Figure 4). There was a significant correlation between both parameters suggesting that differences in the initial slope of CFGE curves (inset of Figure 3) reflect the different number of dsb induced, as proposed above. In conclusion, the number of dsb induced per Gy and Da showed a substantial variation for the tumour cells, but not for normal fibroblasts.

As a novelty, GFGE allows to quantify the retention of DNA fragments in the plug (Dahm-Daphi and Dikomey, 1995). This retention factor, *f*_ret_, depends on cell type, proliferation and presumably other effects. It needs to be known for calculation of the exact number of dsb. For the tumour cells the retention factor *f*_ret_ ranged between 0.11 (HeLa) and 0.4 (MCF7-Bus) (Table 2), which indicates that for HeLa cells only 11% of all fragments with a molecular size below 11.5 Mbp were trapped in the plug in contrast to 40% for MCF7-Bus.

### Rejoining of dsbs

Figure 5A

**Figure 5:**
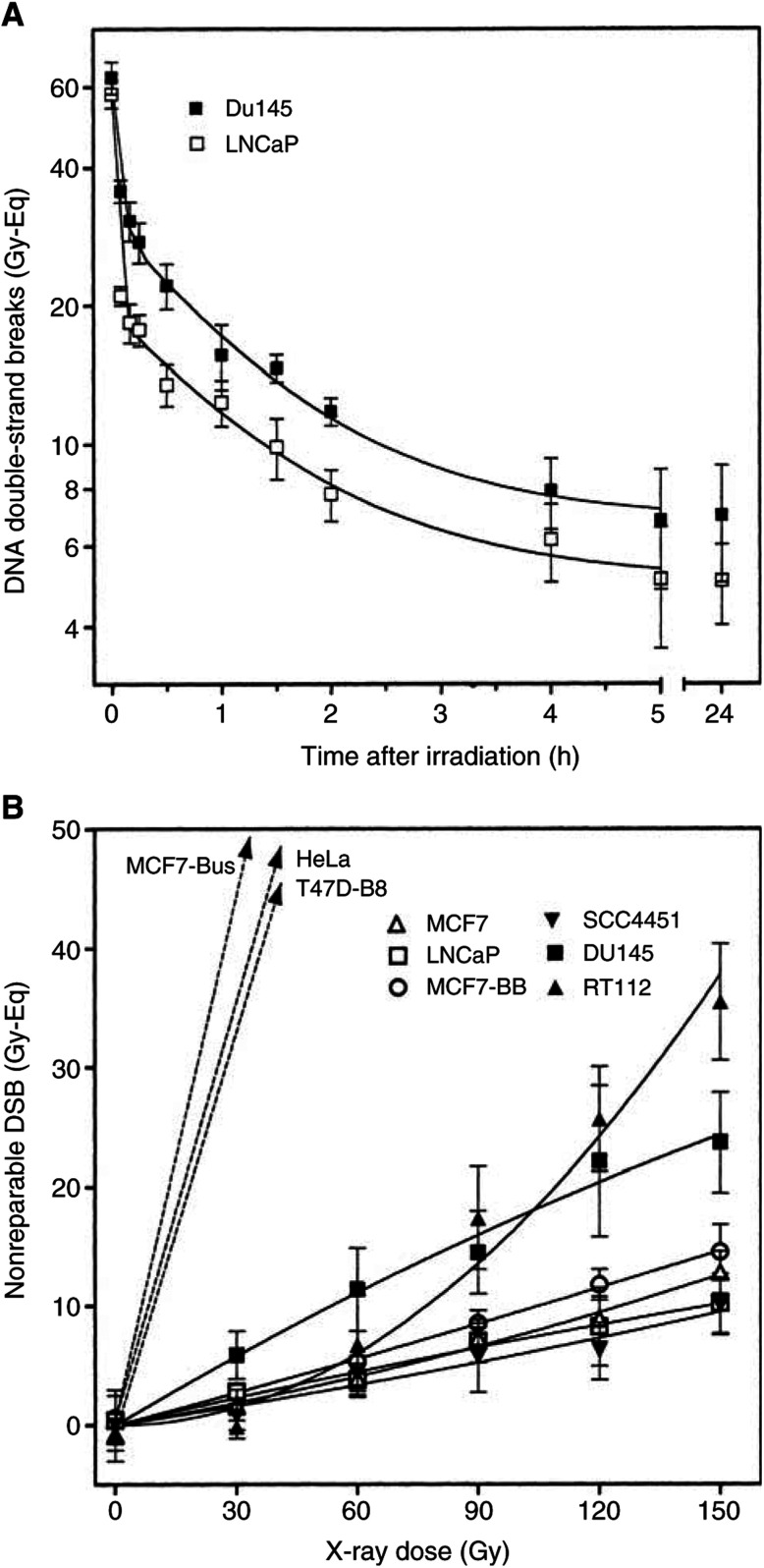
(**A**) Kinetics of dsb repair in DU145 and LNCaP cells. After irradiation with 60 Gy, cells were incubated at 37°C for up to 24 h and dsb were measured by CFGE. The FDR obtained were converted into Gy-equivalents. Data were fitted by nonlinear regression. (**B**) Number of dsb 24 h after irradiation with doses up to 150 Gy. For T47D-B8, MCF7-Bus and HeLa cells, damage measured 24 h after irradiation was higher than the number of dsb initially induced (dashed lines). Data were fitted by nonlinear regression.

shows the repair kinetics of dsb for DU145 and LNCaP. The FDR as obtained from CFGE were converted into Gy-equivalents using the induction curves (Figure 3) for calibration. The repair curves revealed a fast and a slow exponential component with almost identical half-times for both cell lines (*τ*_fast_=3–4 min and *τ*_slow_=90–100 min). These data illustrate that tumour cells are unlikely to process dsb with different kinetics compared to normal cells, which was also observed previously for mouse and rat tumour cells (Dikomey *et al*, 1995, 1998). However, in LNCaP cells more dsb were rejoined with fast kinetics than in DU145 and analogously more dsb remain nonrepaired in DU145 cells.

### Residual dsbs

Residual damage was measured in more detail 24 h after irradiation. In order to record the exact number of dsb, GFGE would be the favorite method; however, the minimum amount DNA released after 24 h cannot be properly resolved. We thus applied CFGE and came up with heterogeneous results (Figure 5B). Four tumour cell lines (SCC4451, LNCaP, MCF7 and MCF7-BB) showed very little residual damage of 8–13 Gy-Eq 24 h after 150 Gy, while in DU145 and RT112 clearly more dsb remained unrepaired. T47D-B8, MCF7-BUS and HeLa cells presented values that even exceeded the initial numbers of dsb induced. The respective gels revealed a DNA smear down to fragment sizes of about 5 kb (not shown). In those cases, the residual dsb rather reflect apoptotic degradation than incomplete repair.

### Cell cycle distribution

**Figure 6:**
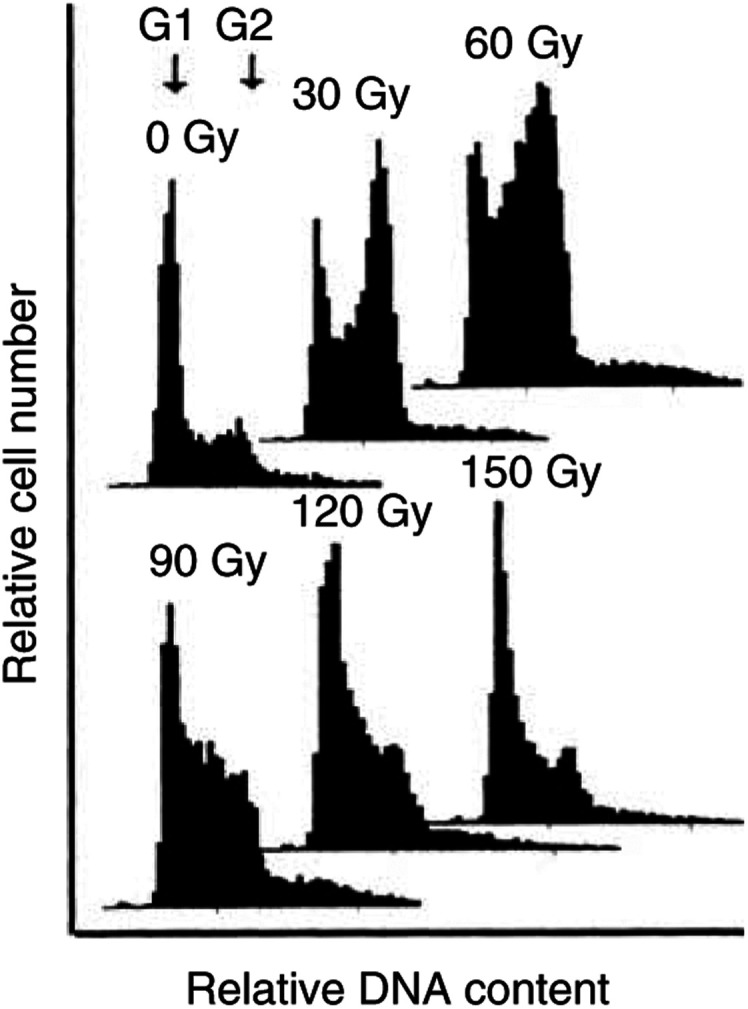
Cell cycle distribution in RT112 cells 24 h after exposure to doses up to 150 Gy. Ethanol-fixed cells were PI stained and subjected to DNA flow cytometry.

Constant-field gel electrophoresis measurements are known to be dependent on the cell cycle distribution. S-phase cells show a smaller FDR than G1 cells even for an identical number of dsb (Dahm-Daphi and Dikomey, 1995). Replication forks are suggested to hinder DNA from migration. DNA flow cytometry of RT112 cells 24 h after irradiation showed a cell cycle distribution varying substantially with dose (Figure 6

). In particular, the fraction of S-phase cells increased up to 72% and declined thereafter. An appreciable variation in cell cycle distribution was also observed for most of the other strains (Table 1). After 150 Gy, some cell lines showed either more (LNCaP, MCF-7, MCF7-Bus) or fewer cells in S phase (T47D-B8, MCF7-BB) than the respective controls. However, even when the cell cycle distribution was the same at 0 and 150 Gy (DU145, SCC4451), a variation might have occurred at other doses, as observed in RT112. In conclusion, the measurement of residual damage is variably influenced by the cell cycle and, thus, do not solely reflect the actual number of dsb.

### Correlation between clonogenic radiosensitivity and initial or residual dsbs

Figure 7

**Figure 7:**
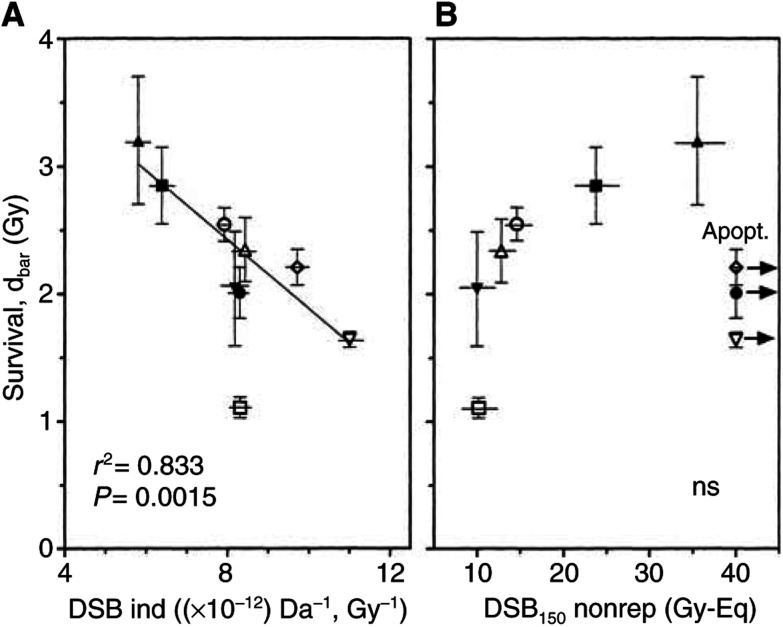
Relationship between dsb and cellular radiosensitivity. (**A**) Number of dsb induced per Gy and per Da (as calculated from Figure 4) and (**B**) number of dsb measured 24 h after 150 Gy (taken from Figure 5B) were plotted *vs* cellular radiosensitivity expressed as *D*_bar_ (from Table 1).

shows the relationship between the clonogenic radiosensitivity and the number of dsb induced (A) or 24 h after irradiation with 150 Gy (B). The cell killing (*D*_bar_) was found to be significantly correlated with the number of induced dsb (*r*^2^=0.833, *P*=0.0015). LNCaP cells were excluded, since the data point was exterior to the range of 2 s.d. However, the correlation was even significant when LNCaP was included (*r*^2^=0.47, *P*=0.041). The data indicate that at least for eight out of nine tumour cells, the variation in sensitivity could result from differences in the initial damage.

The relationship between residual dsb and sensitivity gave a nonsignificant trend. This trend, however, was presumably not meaningful, since an increase in the number of residual dsb was associated with a decrease in sensitivity. In contrast to the initial damage, residual dsb as determined by CFGE appeared not to be an appropriate indicator of the clonogenic radiosensitivity.

## DISCUSSION

The present study was aimed to define the role of initial and residual dsb on the radiosensitivity of human tumour cells.

### Relationship between induced damage and cellular radiosensitivity

For the nine tumuor cell lines tested, the number of dsb induced was found to vary by a factor of 2 from 5.75 to 11.0 × 10^−12^ dsb/Gy/Da (Table 2), which in principle agreed with most previous studies (Kelland *et al*, 1988; McMillan *et al*, 1990; Schwartz *et al*, 1990, 1991; Ruiz de Almodovar *et al*, 1994; Zaffaroni *et al*, 1994; Whitaker *et al*, 1995; Woudstra *et al*, 1998; Eastham *et al*, 2001) using either PFGE or neutral filter elution. Some authors did not mention differences in particular; however, variations were in the same order of magnitude as the above reports (Giaccia *et al*, 1992; Olive *et al*, 1994; McKay and Kefford, 1995). There was only one report (Olive *et al*, 1994) that found differences among six tumour cell lines using neutral filter elution but factually not by PFGE. Our result is important for three reasons. Firstly, the technique used is superior to others (see below) and, second, the variation in damage induction appears to be a property of tumour, but not of normal cells (see below). Most importantly, for eight out of the nine tumour cell lines the variation in the frequency of induced dsb showed a significant correlation with the respective variation in radiosensitivity (Figure 7A). Cell lines with a high number of dsb induced found to be much more sensitive than cell lines with a low number of induced dsb. This was similarly found by Ruiz de Almodovar *et al* (McMillan *et al*, 1990; Ruiz de Almodovar *et al*, 1994; Whitaker *et al*, 1995), other studies showed an insignificant trend (Schwartz *et al*, 1988; Giaccia *et al*, 1992; Zaffaroni *et al*, 1994; McKay and Kefford, 1995; Woudstra *et al*, 1998). In our study, one (LNCaP, Figure 7A) out of the nine cell lines fell off the general relationship between initial damage and cell survival indicating that the cellular radiosensitivity is eventually not only determined by the number of induced dsb but also by other still unknown factors.

The variations in the dsb induction frequency found for tumour cells are most likely due to different chromatin structures. High condensation of chromatin structure and tight DNA–protein association should efficiently protect from oxygen radical attack to DNA, and *vice versa*. Such structural variations among tumour cells have in fact been shown by means of the Halo-assay (Schwartz and Vaughan, 1989; Lynch *et al*, 1991; Woudstra *et al*, 1998). However, direct evidence for chromatin structure being responsible for differences in the dsb induction among tumour cells is lacking yet. It should be noted that the large differences in the dsb induction observed are mainly due to the three extreme values. This may indicate that not all tumour cell lines have an altered chromatin structure.

Contrary to tumour cells, the seven normal fibroblasts tested showed only scarce variation in the number of dsb induced, which confirmed previous results of five and twelve fibroblast lines (Wurm *et al*, 1994; Dikomey *et al*, 2000). Taken all together, it can be assumed that the number of dsb induced varies substantially in tumour cells, but not in normal human cells. The latter might reflect the general interindividual stability of the human genome.

### Graded *vs* CFGE

Most measurements of dsb were performed with neutral filter elution, PFGE or CFGE, which all rely on quantification of overall fragments released from the bulk DNA. The newly applied GFGE has now the advantage to allow the direct determination of the number of dsb. It is shown here, for the first time, that this number was in fact correlated with the initial slope (up to 20 Gy) of the FDR curve of CFGE. However, this does not mean that the initial slope can reliably taken as an indicator for the amount of initial damage. In many cases, the variation in the initial slopes of FDR curves may not be large enough to reveal significant differences between the cell lines. The initial slope further depend on S-phase cells and on the retention factor *f*_ret_. High fractions of replicating cells and high retention values decrease the slope and apparently the number of induced dsb. This might well be the reason why only three out of 13 studies found a relationship between induced damage and tumour cell radiosensitivity (Kelland *et al*, 1988; Schwartz *et al*, 1988, 1990, 1991; McMillan *et al*, 1990; Giaccia *et al*, 1992; Olive *et al*, 1994; Ruiz de Almodovar *et al*, 1994; Zaffaroni *et al*, 1994; McKay and Kefford, 1995; Whitaker *et al*, 1995; Woudstra *et al*, 1998; Eastham *et al*, 2001). In conclusion, GFGE is the preferable method over a standard FDR assay to measure dsb induction in tumour cells.

### Number of induced dsb

The mean dsb induction frequency of all 16 cell lines was 8.1 × 10^−12^ dsb/Gy/Da. (Table 2). This value confirmed our previous results (Dahm-Daphi and Dikomey, 1995; El-Awady *et al*, 2001) and also agreed well with other data (range 8–15 × 10^−12^ dsb/Gy/Da) based on PFGE, from which a number of dsb can be calculated when it is combined with either ^125^I-decay, analysis of fragment size distribution after high doses, or restriction digest (Blöcher and Pohlit, 1982; Ager and Dewey, 1990; Iliakis *et al*, 1991a, 1991b; Lawrence *et al*, 1993; Cedervall *et al*, 1994, 1995; Löbrich *et al*, 1994a, 1994b; Rothkamm and Löbrich, 1999, 2003). Only, Ruiz de Almodovar *et al* (1994) found a much higher induction frequency of about 66 dsb × 10^−12^/Gy/Da.

### Residual dsb

The amount of nonrepaired damage showed a broad variation among the nine tumour cell lines. However, it was shown that apoptosis (Chukhlovin *et al*, 1995) and also cell cycle progression could have an impact on dsb measurements, which means that the residual damage recorded does not exclusively reflect repair capacity. In line with that we did not find a correlation between residual damage and cell survival, which also agreed with other reports (Kelland *et al*, 1988; Olive *et al*, 1994; McKay and Kefford, 1995; Nunez *et al*, 1995; Whitaker *et al*, 1995; Woudstra *et al*, 1996). Surprisingly, four studies found such a correlation but only when the residual damage was measured 1–2 h after irradiation (Schwartz *et al*, 1988, 1990; Giaccia *et al*, 1992; Zaffaroni *et al*, 1994). It may well be that the impact of cell cycle and apoptosis was minimum after such short repair intervals and the data, thus, reflect mainly the amount of dsb induced and initial repair efficiency. Of note, for our data, the association between induced damage and radiosensitivity appears to be close, which means that the impact of the repair capacity should be only marginal.

The recently introduced technique to visualise sites of histone *γ*-H2AX phosphorylation (Rogakou *et al*, 1998) needs much lower doses than gel electropheresis to monitor dsb repair (Rothkamm and Löbrich, 2003) and may thus reduce apoptosis and cell cycle pertubation, although this technique is generally also sensitive to DNA degradation (Rogakou *et al*, 2000) and stalled replication (Ward and Chen, 2001). However, it needs to be shown whether recording of *γ*-H2AX will be an advantage over conventional gel electrophoresis for monitoring residual damage in tumour cells.

In contrast to tumour cells, for normal human and rodent fibroblasts, the variation in cellular sensitivity did not result from differences in the induction, but the repair of dsb (Dahm-Daphi *et al*, 1994; Kiltie *et al*, 1997; Zhou *et al*, 1997a, 1997b; Dikomey *et al*, 1998, 2000). These results suggest that the mechanisms affecting radiosensitivity are different for tumour and normal cell lines.

## CONCLUSION

Tumour cells vary considerably in their amount of induced damage. These differences are most likely due to variations of chromatin structure and they may largely account for tumour cell survival. The number of residual damage was also different among the cell lines studied, but we could now show that those measurements do not only depend on the repair capacity *per se* but also on the cell cycle progression and in some cases on DNA degradation, presumably due to apoptosis.
